# Differentiating second primary lung cancer from pulmonary metastasis in patients of single solitary pulmonary lesion with extrapulmonary tumor using multiparametric analysis of FDG PET/CT

**DOI:** 10.1007/s12149-025-02034-7

**Published:** 2025-03-05

**Authors:** Honghong Liu, Xiaolin Meng, Guanyun Wang, Shulin Yao, Yanmei Wang, Ruimin Wang, Tao Wang

**Affiliations:** 1 Departments of Nuclear Medicine, the First Medical Center, Chinese PLA General Hospital, No.28 Fuxing Road, Beijing, 100853 China; 2 Department of Nuclear Medicine, Beijing Friendship Hospital, Capital Medical University, Beijing, 100050 China; 3 GE Healthcare China, Pudong New Town, Shanghai, 200125 China; 4 Departments of Thoracic Surgery, the First Medical Center, Chinese PLA General Hospital, No.28 Fuxing Road, Beijing, 100853 China

**Keywords:** Multiparameter, Positron emission tomography/computed tomography (PET/CT), Second primary lung cancer (SPLC), Pulmonary metastasis (PM), Radiomics

## Abstract

**Objective:**

Using fluorine-18-fluorodeoxyglucose (^18^F-FDG) positron emission tomography/computed tomography (PET/CT), a multiparametric analysis will be performed in the differential diagnosis of patients with single solitary pulmonary lesion and extrapulmonary malignant tumor to discriminate between a second primary lung cancer (SPLC) and pulmonary metastasis (PM).

**Methods:**

This study retrospectively studied 84 patients with preoperative exams utilizing ^18^F-FDG PET/CT. Using complementing PET/CT parameters, a composite model was developed. A receiver operating characteristic (ROC) analysis assessed the combined model and each independent parameter's differential diagnostic efficacies. Furthermore, this study investigated the improvement in diagnostic efficacy using other metrics, such as integrated discriminatory improvement (IDI) and net reclassification improvement (NRI).

**Results:**

The highest discriminative diagnostic value was obtained by the independent parameters energy (1,039,358.1 [95126.2–1,965,032.2] vs. 92,011.0 [45916.3–365,322.9], *P* = 0.001). In comparison to peak standardized uptake value (SUVpeak), total lesion glycolysis (TLG), energy, lobulation, and spiculation alone, the combined model (addition of these factors) significantly improved the differential diagnostic efficacy of SPLCs and PMs (sensitivity = 76.2%, specificity = 83.8%, area under the curve [AUC] = 0.826) and permitted reclassification using IDI = 0.176 (*P* < 0.001), 0.169 (*P* < 0.001), 0.127 (*P* < 0.001), and categorical NRI = 0.678 (*P* < 0.001), 0.637 (*P* < 0.001), and 0.592 (*P* < 0.001) compared to SUVpeak, TLG and energy separately. DeLong’s test revealed a statistically significant enhancement in ROC when compared to SUVpeak (Z = 2.372, *P* = 0.018), TLG (Z = 2.095, *P* = 0.036), and energy (Z = 2.318, *P* = 0.020).

**Conclusion:**

Combining multiple parameters using ^18^F-FDG PET/CT may further improve distinguishing between SPLCs and PMs in patients with single solitary pulmonary lesion and extrapulmonary malignant tumor.

## Introduction

Solitary pulmonary lesions are often detected in patients with extrapulmonary malignant tumors, and accurate diagnosis of these lesions is critically important because they may affect the selection of an optimum treatment strategy. Secondary primary malignancies may be identified concurrently with main tumors or exclude the underlying cancers’ spread and recurrence afterward. Evidence suggests that the lung is among the most prevalent organs that produce secondary primary malignancies [1, 2]. Between 8 and 14% of lung cancer incidences are second primary lung cancers (SPLCs), which are thought to be the main factor influencing cancer survivors' future life expectancy [2].

Postoperative hematogenous metastasis is the most prevalent cause of pulmonary metastasis (PM) after curative therapy [3]. As a result, precise diagnosis of solitary pulmonary lesions identified in patients with malignant tumors is essential for determining an optimal therapeutic management strategy to enhance prognosis.

The gold standard for diagnosing SPLC or PM is still pathological investigation, which is dependent on invasive techniques or surgical resection and is frequently not possible because of the patient’s unwillingness or poor clinical state. The predominant assessment of pulmonary lesions utilizes conventional imaging techniques, including X-ray and computed tomography (CT), which offer restricted diagnostic insights. Lung cancer is among the many malignancies for which fluorine-18-fluorodeoxyglucose (^18^F-FDG) positron emission tomography/computed tomography (PET/CT) is frequently utilized for diagnosis, therapy response assessment, and outcome prediction [4]. Still, a significantly larger quantity of information is inside the images, necessitating alternative analyzing techniques.

Radiomics, the high-throughput extraction of numerous features from medical images, is a field where multiparameter analysis is widely employed in diagnostic imaging modalities [5, 6]. Radiomic characteristics encompass image-derived descriptors that objectively represent the form, size, volume, and texture of cancers or normal tissue [7]. Predictive models developed using multiparameter analysis can be used for various clinical research tasks, including prognosis, therapy response assessment, and preoperative diagnosis compared to traditional independent variables [8]. Therefore, the intertumoral heterogeneity identified by ^18^F-FDG PET/CT is quantitatively assessed utilizing various imaging features based on radiomic analysis [9, 10].

Although the correlation between single solitary pulmonary nodule and extrapulmonary primary tumor is addressed in the literature [1, 11], many associated problems require further research. For example, few studies, to our knowledge, systematically explore the characteristics of single solitary pulmonary lesion with an extrapulmonary malignant tumor on PET/CT imaging, particularly when subjected to multiparameter analysis [12–14]. This study employed multiparameter analysis utilizing ^18^F-FDG PET/CT to independently and collectively assess the efficiency of differentiating single solitary pulmonary lesion with an extrapulmonary malignant tumor.

## Materials and methods

### Patients

We examined PET/CT scans of 84 patients treated at our institution between January 2015 and December 2022 who had a single pulmonary lesion with extrapulmonary malignant tumors. The average age was 64.9 ± 9.3 years (range = 43 to 82 years). After receiving ^18^F-FDG PET/CT scans, all patients received a histological examination within two weeks of the procedure. Lesion samples were acquired by CT-guided percutaneous core biopsy (n = 45) or surgery (n = 39). The following were the requirements for inclusion: (1) before the discovery of isolated pulmonary tumors and without any indication of additional distant metastases, a history of an extrapulmonary malignant tumor; (2) had ^18^F-FDG PET/CT before therapy; and (3) pathological diagnosis of SPLCs or PMs based on histological findings. The following were the requirements for exclusion: (1) low-quality PET/CT scans of ^18^F-FDG and (2) no or incomplete clinical data.

### PET/CT examination

A Discovery 710 (GE Healthcare) was used to perform an integrated whole-body PET/CT scan on patients who had fasted for at least 6 to 8 h before their scans. After injecting ^18^F-FDG (radiochemical purity > 95%, 3.70–4.44 MBq/kg), imaging was carried out one hour later. Before imaging, all patients were instructed to empty their bladders. The proximal thigh to the base of the brain was scanned for the CT and PET scans. The following were the low-dose CT parameters: pitch, 1; rotation, 0.8; layer thickness, 3.75 mm; voltage, 120 kV; and current, 100 mA. The PET settings were 3D mode, 3 min/bed, 6–7 beds/person, three iterations, 21 subsets, and a Gaussian filter half-height width of 4.0 mm. An ordered subset expectation maximization approach was used to reassemble the pictures with CT attenuation correction. A postprocessing workstation was used to merge the images after they had been shown separately.

### Image analyses

Two experienced nuclear medicine specialists independently assessed the ^18^F-FDG PET/CT scans. To establish a 3D volume of interest (VOI), a 2D region of interest (ROI) was manually drawn around the lesion boundary on each layer of the transaxial CT scan. The CT lesion features were visually analyzed: location, length, shape (round-oval or lobulated), and margin (spiculated or smooth). Furthermore, we obtained the standard PET/CT metabolic parameters, such as maximum standardized uptake normalized to lean body mass (SULmax), total lesion glycolysis (TLG), peak standardized uptake normalized to lean body mass (SULpeak), mean standardized uptake normalized to lean body mass (SULmean), and maximum standardized uptake value (SUVmax), peak standard uptake value (SUVpeak), and mean standard uptake value (SUVmean).

Using LIFEx software (www.lifexsoft.org/index), the pulmonary nodule or mass was identified on axial PET images [15]. A 3D segmentation was automatically depicted using a fixed threshold = 40% SUVmax and then manually corrected by radiologists uninformed of patients' pathological results. Following the Image Biomarker Standardization Initiative, radiomic characteristics were extracted from each derived PET VOI using the PyRadiomics module in Python 3.8.1 [16]. The following are the 107 radiomic characteristics that we extracted: 25 first order features, 14 shape features,14 Gy level dependence matrix (GLDM) features, 16 Gy level size zone matrix (GLSZM) features, 16 Gy level run length matrix (GLRLM) features, and 22 Gy level co-occurrence matrix (GLCM) features. Subsequent analysis was performed for radiomic features using variance analysis (*P* < 0.05).

### Statistical analyses

R (version 4.0.2, Bell Laboratories, USA) and SPSS version 21.0 (Inc., Armonk, New York, USA) were used for all analyses. Qualitative variables are represented as C counts and percentages (n [%]), which are then assessed using the chi-square test. The mean ± SD summarizes normally distributed continuous variables, while the median within the interquartile range (IQR) summarizes skewed distributed continuous variables. The distribution of baseline features and PET/CT metabolic parameters between the two groups (SPLC and PM) was investigated using the Student t-test (with variances adjusted using Levene’s test) and the chi-square test. The area under the receiver operating characteristic (ROC) curve was calculated to assess the predictive value of ^18^F-FDG PET metabolic parameters. Calculations were made to determine the negative predictive value (NPV), positive predictive value (PPV), specificity, and sensitivity. In order to compare the ^18^F-FDG metabolic parameters and radiomic features between SPLCs from PMs with the highest area under the curve (AUC), we chose to include the lobation and spiculation characteristics as well as data discrepancies with *P* < 0.01 in the diagnostic model. A diagnostic model was built to differentiate SPLC from PM using multivariate logistic regression analysis. Diagnostic models with and without metabolic data and PET/CT radiomic characteristics were compared using the net reclassification improvement (NRI) and integrated discriminatory improvement (IDI). A statistically significant difference was defined as *P* < 0.05.

## Results

### CT imaging data and baseline patient features

Among the 84 patients selected for this study, 38 and 42 were included in the SPLC and PM groups, respectively. Because of the small sample size, two lung carcinoids, one pulmonary hamartoma and one pulmonary sclerosing pneumocytoma were deleted when conducting statistical analysis. The original primary tumors included 42 gastrointestinal cancers, 6 breast cancers, 6 uterine cancers (4 endometrial cancers and 2 cervical cancers), 5 esophageal cancers, 4 renal cell carcinomas, 3 pancreatic cancers, 3 bladder cancers, 2 liver cancers, 2 prostate cancers, 2 thyroid cancers, 2 retroperitoneal leiomyosarcomas, 1 cholangiocarcinoma, 1 tongue cancer, and 1 laryngeal cancer. The ages of the SPLC and PM patients were 68.0 years (range 60.5–73.5 years) and 60.0 years (range 54.0–71.0 years) (*P* = 0.036), respectively. Furthermore, in the two respective groups, lobulation (n = 36 [97.3%] vs. n = 33 (78.6%], *P* = 0.016) and spiculation (n = 17 [45.9%] vs n = 8 [19.0%], *P* = 0.010) differed significantly. Although the difference was not statistically significant (*P* = 0.054), the average diameters of the SPLCs were higher than those of the PMs ([31.7 ± 13.5]mm vs [25.6 ± 14.1]mm). The groups exhibited no significant differences in sex or interval time (Table 1).

**Table 1:** Demonstrates the disparities in baseline features and PET/CT parameters between patients with SPLC and PM

Variable	SPLC (*n* = 38)	PM (*n* = 42)	*P*
Baseline characteristics			
Age (years)	68.0 (60.5–73.5)	60.0 (54.0–71.0)	0.036
Male: Female (n, %) Interval time (months)	21 (56.8%): 16 (43.2%) 7.0 (0.0–45.5)	26(61.9%):16 (38.1%) 13.5 (2.3–31.3)	0.642 0.861
CT imaging data of PET/CT			
Diameter (mm)	31.7 ± 13.5	25.6 ± 14.1	0.054
Lobation (n, %)	36 (97.3%)	33 (78.6%)	0.016
Spiculation (n, %)	17 (45.9%)	8 (19.0%)	0.010
Metabolism parameters of PET/CT			
SUVmax	10.8 (4.6–18.8)	7.6 (4.7–11.0)	0.016
SUVmean	6.6 (2.8–11.2)	4.7 (2.8–6.7)	0.020
SUVpeak	9.1 (3.4–15.1)	5.9 (2.8–8.1)	0.003
SULmax	8.1 (3.9–14.2)	6.1 (3.6–8.3)	0.012
SULmean	5.2 (2.3–8.5)	3.7 (2.2–5.3)	0.017
SULpeak	7.1 (2.8–11.2)	4.5 (2.2–6.6)	0.005
TLG	60.6 (16.7–184.8)	18.0 (10.8–39.7)	0.003
Radiomics parameters of PET/CT			
90 Percentile	8.1 (6.6–13.8)	6.4 (3.9–8.8)	0.009
Energy	1,039,358.1 (95,126.2–965,032.2)	92,011.0 (45,916.3–365,322.9)	0.001
Interquartile Range	2.9 (2.0–4.8)	1.8 (1.1–3.2)	0.016
Maximum	11.3 (8.4–19.0)	8.2 (5.0–11.7)	0.009
Mean	5.4 (3.3–8.0)	3.9 (2.7–5.7)	0.014
Mean Absolute Deviation	1.7 (1.1–2.8)	1.1 (0.6–1.8)	0.013
Median	5.0 (3.0–6.8)	3.6 (2.7–5.1)	0.034
Range	9.5 (6.6–17.0)	6.4 (3.7–10.6)	0.010
Robust Mean Absolute Deviation	1.2 (0.8–2.0)	0.8 (0.4–1.3)	0.013
Root Mean Squared	5.7 (4.0–9.2)	4.3 (2.8–6.1)	0.012
Variance	4.2 (1.8–12.0)	1.8 (0.5–4.9)	0.015
Minor Axis Length	28.8 ± 9.2	22.6 ± 9.7	0.005

PET/CT: positron emission tomography/computed tomography; SPLC: second primary lung cancer; PM: pulmonary metastasis; SUVmax: maximum standardized uptake value; SUVmean: mean standard uptake value; SUVpeak: peak standard uptake value; SULmax: maximum standardized uptake normalized to lean body mass; SULmean: mean standardized uptake normalized to lean body mass; SULpeak: peak standardized uptake normalized to lean body mass; TLG: total lesion glycolysis

### Comparison of ^18^F-FDG PET parameters between SPLCs and PMs

**Fig. 1:**
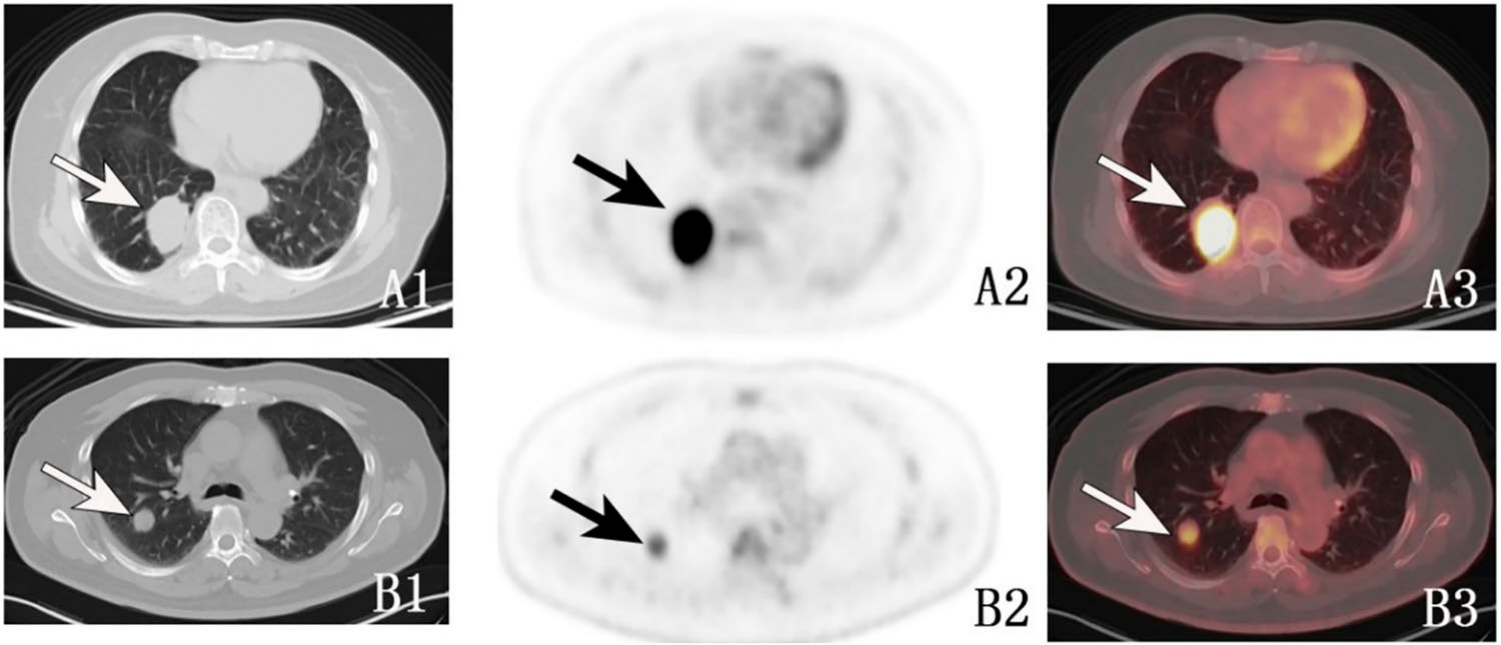
**A** The axial PET/CT images of a 71-year-old female patient, 96 months post-resection of cervical cancer, reveal a solitary lung mass in the right lower lobe (**A1** arrow: axial CT image, dimensions: 30 mm × 39 mm) and significant hypermetabolism of glycose (**A2** and **A3** arrows: axial PET and PET/CT images, SUVpeak: 15.8, TLG: 193.6). The pathological diagnosis was SPLC. **B** The axial PET/CT images of a 50-year-old male patient with colon cancer. Simultaneously, a solitary lung nodule was found in the right upper lobe (**B1** arrow: the axial CT image, size: 19 mm × 20 mm) as well as hypermetabolism of glycose (**B2** and **B3** arrows: the axial PET and PET/CT images, SUVpeak: 3.8, TLG: 12.6). The pathological diagnosis was PM

**Fig. 2:**
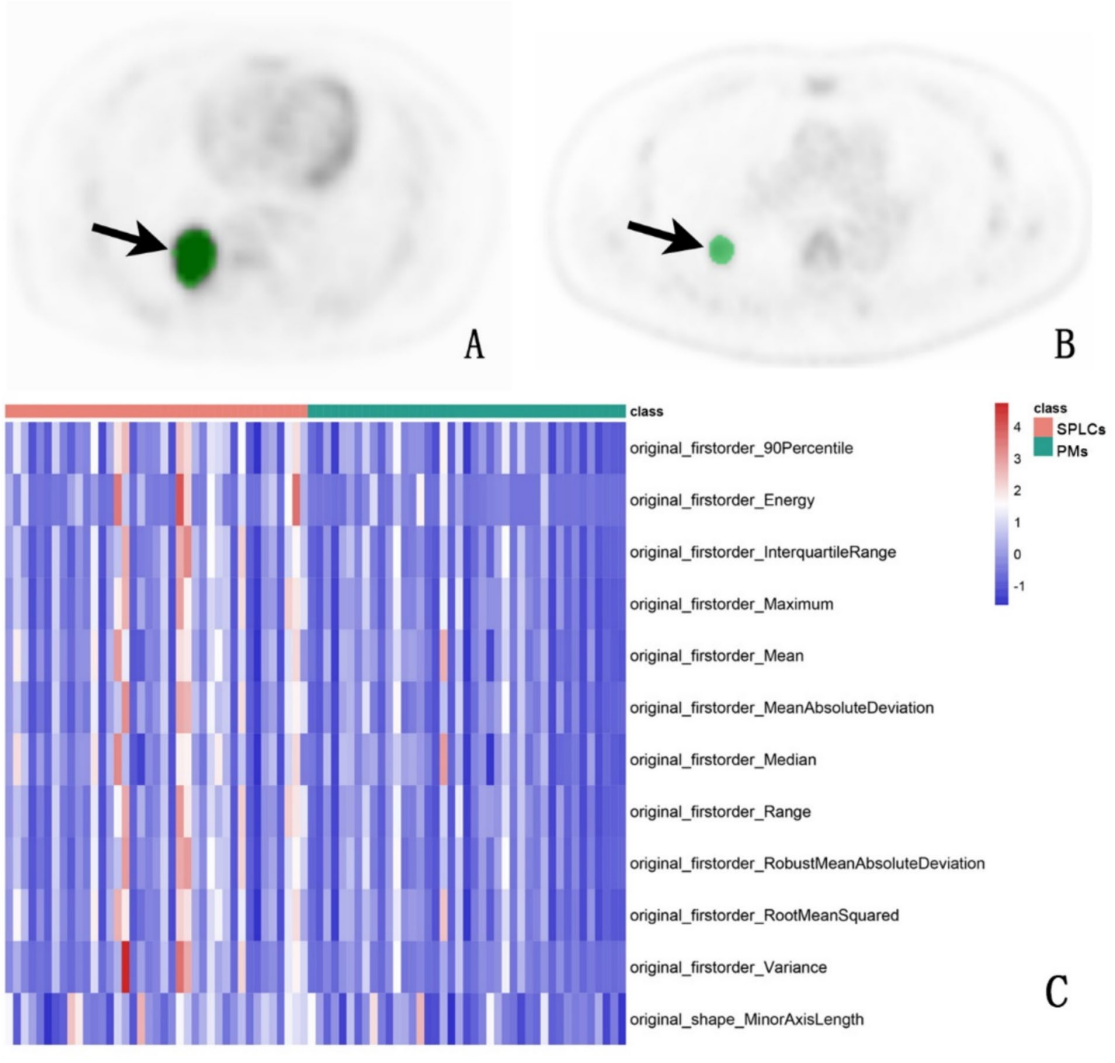
**A** The axial PET image of the radiomic analysis of a patient with SPLC. **B** The axial PET image of the radiomic analysis of a different patient with PM. **C** Heat map of PET radiomic parameters of SPLCs and PMs

SUVmax, SUVmean, SUVpeak, SULmax, SULmean, SULpeak, and TLG were significantly higher in patients with SPLCs than patients with PMs. In particular, SUVpeak (9.1 [3.4–15.1] vs. 5.9 (2.8–8.1), *P* = 0.003) and TLG (60.6 [16.7–184.8] vs 18.0 (10.8–39.7), *P* = 0.003) were higher than in patients with PMs (Fig. 1). For radiomic parameters, 12 out of 107 radiomic features (*P* < 0.05) were selected for subsequent analysis (Table 1). This study found that patients with SPLCs had significantly higher radiomic parameters of ^18^F-FDG PET (root mean squared, robust mean absolute deviation, range, median, mean absolute deviation, mean, interquartile range, maximum, energy, 90th percentile, variance, and minor axis length) than patients with PMs, with energy being the most significant (1,039,358.1 [95126.2–1,965,032.2] vs. 92,011.0 [45916.3–365,322.9], *P* = 0.001) (Fig. 2).

### Diagnostic efficacy of ^18^F-FDG PET parameters and the model

ROC analysis indicated that energy achieved the highest discriminative diagnostic value (cut-off = 996,258.5 [sensitivity = 0.929; 95% CI: 0.794–0.981], specificity = 0.514 [95% CI: 0.347–0.678], AUC = 0.714 [95% CI: 0.594–0.833], PPV = 0.684 [95% CI: 0.546–0.797], and NPV = 0.864 [95% CI: 0.640–0.964]).

**Fig. 3:**
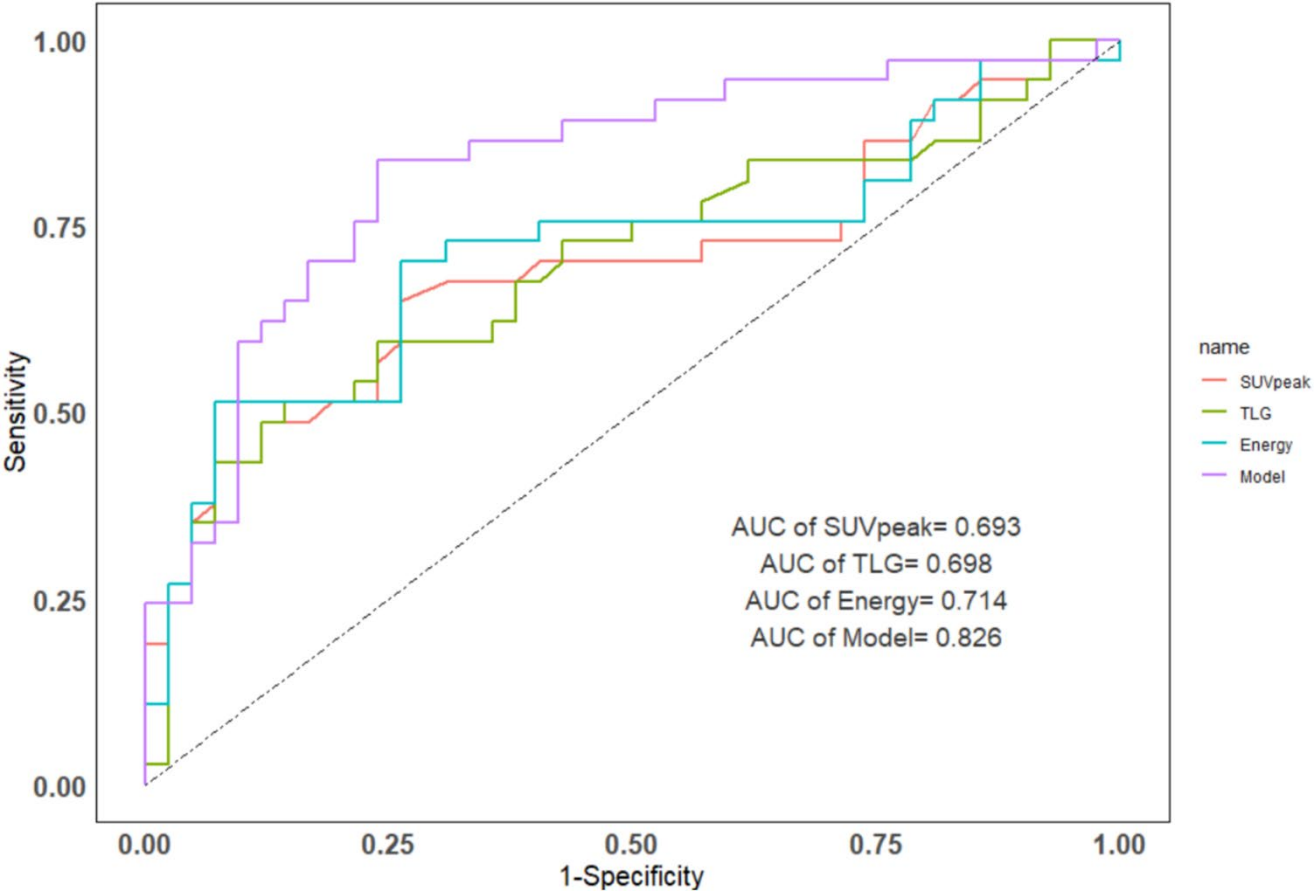
ROC curves of the top 3 independent ^18^F-FDG PET variables and the combined model

To improve diagnostic performance, we constructed a logistic regression model incorporating metabolic, volume, texture parameters, and CT features. The combined model yielded AUC = 0.826 (95% CI: 0.732–0.920), sensitivity = 0.762 (95% CI: 0.602–0.874), specificity = 0.838 (95% CI: 0.673–0.932), PPV = 0.842 (95% CI: 0.681–0.934), and NPV = 0.756 (95% CI: 0.594–0.871) (Fig. 3 and Table 2).

**Table 2:** The differential diagnostic efficiency between SPLC and PM for the top three independent ^18^F-FDG PET parameters and the model

Parameters	Cut-off	AUC (95%CI)	Sensitivity (95%CI)	Specificity (95%CI)	PPV (95%CI)	NPV (95%CI)
SUVpeak	7.6	0.693 (0.572–0.814)	0.738 (0.577–0.856)	0.649 (0.474–0.793)	0.705 (0.546–0.827)	0.686 (0.506–0.826)
TLG	58.6	0.698 (0.578–0.817)	0.857 (0.708–0.941)	0.514 (0.347–0.678)	0.667 (0.524–0.785)	0.760 (0.545–0.898)
Energy	996,258.5	0.714 (0.594–0.833)	0.929 (0.794–0.981)	0.514 (0.347–0.678)	0.684 (0.546–0.797)	0.864 (0.640–0.964)
Model*	–	0.826 (0.732–0.920)	0.762 (0.602–0.874)	0.838 (0.673–0.932)	0.842 (0.681–0.934)	0.756 (0.594–0.871)

*SUVpeak plus TLG plus Energy plus Lobation plus Spiculation

AUC: area under the curve; PPV: positive predictive value; NPV: negative predictive value

In comparison to the three highest independent ^18^F-FDG PET parameters (SUVpeak, TLG, and energy) individually, the integrated model (which includes SUVpeak, TLG, energy, lobulation and spiculation) facilitated a significant reclassification, demonstrated by IDI values of 0.176 (95% CI: 0.094–0.258, *P* < 0.001), 0.169 (95% CI: 0.085–0.252, *P* < 0.001), and 0.127 (95% CI: 0.052–0.202, *P* < 0.001), as well as categorical NRI values of 0.678 (95% CI: 0.390–0.967, *P* < 0.001), 0.637 (95% CI: 0.400–0.947, *P* < 0.001), and 0.592 (95% CI: 0.323–0.961, *P* < 0.001). The DeLong’s test showed that compared with SUVpeak (Z = 2.372, *P* = 0.018), TLG (Z = 2.095, *P* = 0.036), and energy (Z = 2.318, *P* = 0.020) alone, the model achieved more significant differences in ROC (Table 3).

**Table 3:** Separate comparisons of SUVpeak, TLG, and Energy of the model using NRI, IDI, and DeLong's tests

Variable	DeLong's test		IDI	95%CI	*P*	NRI	95%CI	*P*
	*Z*	*P*						
Model* vs. SUVpeak	2.372	0.018	0.176	0.094–0.258	< 0.001	0.678	0.390–0.967	< 0.001
Model vs. TLG	2.095	0.036	0.169	0.085–0.252	< 0.001	0.637	0.400–0.947	< 0.001
Model vs. Energy	2.318	0.020	0.127	0.053–0.202	< 0.001	0.592	0.323–0.961	< 0.001

*SUVpeak plus TLG plus Energy plus Lobation plus Spiculation

NRI: net reclassification improvement; IDI: integrated discrimination improvement

## Discussion

After the first diagnosis, the chance of getting a second primary cancer rises over time. The risk of developing a second primary non-small cell lung cancer in patients between the ages of 60 and 64 is roughly 10.9%. It may rise steadily over time without declining [17]. The mean age of patients with SPLC exceeded that of patients with PM, which may be attributable to the heightened vulnerability of older individuals to lung cancer. Furthermore, younger patients, in comparison, may have more highly invasive malignant tumors, advanced staging, and a greater likelihood of pulmonary metastasis. Presenting a preliminary judgment of whether cancer is an SPLC or PM is therefore critically important for patients with single solitary pulmonary lesion and extrapulmonary malignant tumor because the respective treatment strategies may completely differ. However, due to the high risk and difficulty of biopsy, particularly for patients with malignant tumors, it is critically important to achieve a differential diagnosis through noninvasive imaging techniques.

A popular imaging method for examining isolated pulmonary lesions is CT. According to the findings, lobulation and spiculation are more indicative of primary lung cancer, which is in line with earlier studies [18]. For the diagnosis, staging, assessment of therapy response, recurrence detection, and prognostic prediction of various cancers, the ^18^F-FDG PET/CT is a crucial imaging technique [9]. Clinical oncology frequently uses prominent ^18^F-FDG PET/CT metrics such as SUV derivatives, TLG and MTV (metabolic tumor volume) [10, 19–21]. However, there are only a limited number of published studies that utilized PET/CT for the differential diagnosis of individuals with single solitary pulmonary lesion and extrapulmonary malignant tumor [13, 14, 20], especially lacking radiomics related analysis.

Incorporation of FDG by malignant tumors is associated with their aggressiveness and prognoses, and numerous PET parameters reflect FDG uptake. Consequently, semiquantitative metrics of ^18^F-FDG uptake (e.g., SUVs) by malignant tumors are widely used for diagnosis and clinical trials of tumor therapies. For example, SUVpeak is a more accurate and robust parameter compared with SUVmax [22], and when SUVpeak is combined with SUVmax, improved reproducibility and accurate quantitation are achieved when applied to the clinic [22]. Furthermore, TLG, which integrates the metabolically active tumor volume with FDG uptake, is significantly associated with clinical outcomes [9]. This study showed that the SPLC group had considerably more significant levels of several PET/CT measures, particularly SUVpeak and TLG. The present results show that these measures were superior to visual analysis in distinguishing between SPLCs and PMs, which probably helps with treatment plan implementation and prognostication.

Radiomics primarily enhances patient management by predicting illness types, survival rates, and treatment success [23]. Lung lesions are systematically assessed and monitored using standard chest CT imaging, whereas ^18^F-FDG PET/CT is utilized to evaluate particular lung lesions. Comprehensive assessments of the diagnostic efficacy of CT-derived radiomics surpass conventional metrics being assessed [24, 25]. The metabolic findings derived from ^18^F-FDG PET demonstrate certain benefits over morphological data acquired only from CT [25–27]. For instance, Zhang et al. [8] showed that, while not statistically significant, the AUC for ^18^F-FDG PET-derived radiomics was higher compared to CT (0.874 ± 0.081 vs 0.820 ± 0.053, respectively).

Established prediction models emphasizing computable imaging features distinguish between benign, malignant, and inflammatory pulmonary conditions [28, 29]. For instance, compared to SUVmax alone, textural analysis of radiotracer uptake values significantly improves specificity [30]. A retrospective texture analysis of a substantial patient cohort (n = 534) was employed to differentiate between metastatic and primary lung lesions. It demonstrated that radiomic features derived from CT and PET datasets could effectively distinguish between them [31]. These results show that a diagnostic model based on the ^18^F-FDG PET metabolic parameters SUVpeak and TLG, radiomics parameter (energy), and CT imaging data (lobation and spiculation) effectively helped differentiate SPLC from PM. The findings indicate that integrating conventional and radiomic parameters of ^18^F-FDG PET/CT enhanced the distinction between SPLC and PM (AUC = 0.826). Furthermore, a potential clinically significant use of radiomics takes advantage of its noninvasive nature, hopefully facilitating its use as a substitute for puncture biopsy. Moreover, ^18^F-FDG PET can distinguish initial pulmonary carcinoma from metastatic tumors [31].

Our study has some limitations. First, its small sample size may bias the interpretations of the accuracies of statistical values as well as the clinical significance of the diagnostic model. Secondly, some participants were eliminated from the study if their ^18^F-FDG PET/CT outcomes were not corroborated due to their refusal or worsening clinical state, leading to their disqualification from invasive diagnostic procedures or surgery. Third, benign findings were not analyzed. The diagnostic model may become statistically biased as a result of these limitations. Fourth, patients with a range of primary extrapulmonary cancers were included; the morphologies of these cancers can vary. Establishing the value of the diagnostic model for patients with single solitary pulmonary lesion with single type of primary malignancy requires further study.

## Conclusions

In summary, numerous metabolic characteristics of ^18^F-FDG PET/CT exhibited variable diagnostic efficacies in distinguishing SPLCs from PMs of patients with single solitary pulmonary lesion and extrapulmonary malignant tumor. The accuracy of differential diagnosis will probably be further increased by combining conventional and radiomic characteristics.

## Data Availability

The datasets used and analysed during the current study are available from the corresponding author on reasonable request.

## References

[1] Ge J, Gou HF, Chen Y, et al. Clinical characteristics of patients with solitary pulmonary mass after radical treatment for primary cancers: pulmonary metastasis or second primary lung cancer? Cancer Invest. 2013;31(6):397–403. 23758185 10.3109/07357907.2013.800092

[2] Araujo-Filho JAB, Halpenny D, McQuade C, et al. Management of pulmonary nodules in oncologic patients: AJR expert panel narrative review. AJR Am J Roentgenol. 2021;16(6):1423–31. 10.2214/AJR.20.24907 33355489

[3] Shin JW, Lee SI, Moon HY. Significance of follow-up in detection of pulmonary metastasis of colorectal cancer. J Korean Soc Coloproctol. 2010;26(4):293–7. 21152232 10.3393/jksc.2010.26.4.293 PMC2998013

[4] Manafi-Farid R, Askari E, Shiri I, et al. [^18^F] FDG-PET/CT radiomics and artificial intelligence in lung cancer: technical aspects and potential clinical applications. Semin Nucl Med. 2022;52(6):759–80. 35717201 10.1053/j.semnuclmed.2022.04.004

[5] Lambin P, Rios-Velazquez E, Leijenaar R, et al. Radiomics: extracting more information from medical images using advanced feature analysis. Eur J Cancer. 2012;48(4):441–6. 22257792 10.1016/j.ejca.2011.11.036 PMC4533986

[6] Hatt M, Tixier F, Visvikis D, et al. Radiomics in PET/CT: more than meets the eye? J Nucl Med. 2017;58(3):365–6. 27811126 10.2967/jnumed.116.184655

[7] Avanzo M, Stancanello J, Pirrone G, et al. Radiomics and deep learning in lung cancer. Strahlenther Onkol. 2020;196(10):879–87. 32367456 10.1007/s00066-020-01625-9

[8] Zhang Y, Cheng C, Liu Z, et al. Radiomics analysis for the differentiation of autoimmune pancreatitis and pancreatic ductal adenocarcinoma in ^18^F-FDG PET/CT. Med Phys. 2019;46(10):4520–30. 31348535 10.1002/mp.13733

[9] Lee JW, Lee SM. Radiomics in oncological PET/CT: clinical applications. Nucl Med Mol Imaging. 2018;52(3):170–89. 29942396 10.1007/s13139-017-0500-y PMC5995782

[10] Wang G, Dang H, Yu P, et al. Multiparameter analysis using ^18^F-FDG PET/CT in the differential diagnosis of pancreatic cystic neoplasms. Contrast Media Mol Imaging. 2021;7:6658644. 10.1155/2021/6658644 PMC8046553 33880111

[11] Quint LE, Park CH, Iannettoni MD. Solitary pulmonary nodules in patients with extrapulmonary neoplasms. Radiology. 2000;217(1):257–61. 11012454 10.1148/radiology.217.1.r00oc20257

[12] Lim CH, Park SB, Kim HK, et al. Clinical value of surveillance ^18^F-fluorodeoxyglucose PET/CT for detecting unsuspected recurrence or second primary cancer in non-small cell lung cancer after curative therapy. Cancers (Basel). 2022;14(3):632. 35158900 10.3390/cancers14030632 PMC8833387

[13] Cegla P, Scibisz-Dziedzic K, Witkowska K, et al. Detection of a second primary cancer in a ^18^F-fluorocholine PET/CT- multicentre retrospective analysis on a group of 1345 prostate cancer patients. Nucl Med Rev Cent East Eur. 2022;25(1):25–30. 35137934 10.5603/NMR.a2022.0006

[14] Ji Y, Wang Y, Zheng JS, et al. Value of ^18^F-FDG PET/CT combined with lung HRCT in diagnosis of solitary pulmonary intravascular metastasis. Contrast Media Mol Imaging. 2022;21:8968855. 10.1155/2022/8968855 PMC8885262 35280706

[15] Nioche C, Orlhac F, Boughdad S, et al. LIFEx: a freeware for radiomic feature calculation in multimodality imaging to accelerate advances in the characterization of tumor heterogeneity. Cancer Res. 2018;78(16):4786–9. 29959149 10.1158/0008-5472.CAN-18-0125

[16] Zwanenburg A, Vallières M, Abdalah MA, et al. The image biomarker standardization initiative: standardized quantitative radiomics for high-throughput image-based phenotyping. Radiology. 2020;295(2):328–38. 32154773 10.1148/radiol.2020191145 PMC7193906

[17] Thakur MK, Ruterbusch JJ, Schwartz AG, et al. Risk of second lung cancer in patients with previously treated lung cancer: analysis of Surveillance, Epidemiology, and End Results (SEER) data. J Thorac Oncol. 2018;13(1):46–53. 28989038 10.1016/j.jtho.2017.09.1964 PMC6108174

[18] Liang TI, Lee EY. Pediatric pulmonary nodules: imaging guidelines and recommendations. Radiol Clin North Am. 2022;60(1):55–67. 34836566 10.1016/j.rcl.2021.08.004

[19] Watabe T, Tatsumi M, Watabe H, et al. Intratumoral heterogeneity of F-18 FDG uptake differentiates between gastrointestinal stromal tumors and abdominal malignant lymphomas on PET/CT. Ann Nucl Med. 2012;26(3):222–7. 22187313 10.1007/s12149-011-0562-3

[20] Ghossein J, Gingras S, Zeng W. Differentiating primary from secondary lung cancer with FDG PET/CT and extra-pulmonary tumor grade. J Med Imaging Radiat Sci. 2023;54(3):451–6. 37355362 10.1016/j.jmir.2023.05.045

[21] Cegla P, Filipczuk A, Cholewinski W. Potential use of [^18^F] FDG heterogeneity in discrimination of two different synchronous primary tumors. Rep Pract Oncol Radiother. 2023;28(3):433–4. 37795392 10.5603/RPOR.a2023.0038 PMC10547406

[22] Akamatsu G, Ikari Y, Nishida H, et al. Influence of statistical fluctuation on reproducibility and accuracy of SUVmax and SUVpeak: a phantom study. J Nucl Med Technol. 2015;43(3):222–6. 26271802 10.2967/jnmt.115.161745

[23] Anan N, Zainon R, Tamal M. A review on advances in ^18^F-FDG PET/CT radiomics standardisation and application in lung disease management. Insights Imaging. 2022;13(1):22. 35124733 10.1186/s13244-021-01153-9 PMC8817778

[24] Beig N, Khorrami M, Alilou M, et al. Perinodular and intranodular radiomic features on lung CT images distinguish adenocarcinomas from granulomas. Radiology. 2019;290(3):783–92. 30561278 10.1148/radiol.2018180910 PMC6394783

[25] Manafi-Farid R, Karamzade-Ziarati N, Vali R, et al. 2-[^18^F] FDG PET/CT radiomics in lung cancer: an overview of the technical aspect and its emerging role in management of the disease. Methods. 2021;188:84–97. 32497604 10.1016/j.ymeth.2020.05.023

[26] Kang F, Mu W, Gong J, et al. Integrating manual diagnosis into radiomics for reducing the false positive rate of ^18^F-FDG PET/CT diagnosis in patients with suspected lung cancer. Eur J Nucl Med Mol Imaging. 2019;46(13):2770–9. 31321483 10.1007/s00259-019-04418-0

[27] Zhang R, Zhu L, Cai Z, et al. Potential feature exploration and model development based on ^18^F-FDG PET/CT images for differentiating benign and malignant lung lesions. Eur J Radiol. 2019;121: 108735. 31733432 10.1016/j.ejrad.2019.108735

[28] Suo S, Cheng J, Cao M, et al. Assessment of heterogeneity difference between edge and core by using texture analysis: differentiation of malignant from inflammatory pulmonary nodules and masses. Acad Radiol. 2016;23(9):1115–22. 27298058 10.1016/j.acra.2016.04.009

[29] Balagurunathan Y, Schabath MB, Wang H, et al. Quantitative imaging features improve discrimination of malignancy in pulmonary nodules. Sci Rep. 2019;9(1):8528. 31189944 10.1038/s41598-019-44562-z PMC6561979

[30] Chen S, Harmon S, Perk T, et al. Diagnostic classification of solitary pulmonary nodules using dual time ^18^F-FDG PET/CT image texture features in granuloma-endemic regions. Sci Rep. 2017;7(1):9370. 28839156 10.1038/s41598-017-08764-7 PMC5571049

[31] Kirienko M, Cozzi L, Rossi A, et al. Ability of FDG PET and CT radiomics features to differentiate between primary and metastatic lung lesions. Eur J Nucl Med Mol Imaging. 2018;45(10):1649–60. 29623375 10.1007/s00259-018-3987-2

